# Focused Ultrasound‐Induced Mechanical Ablation Affects the Carbohydrate Metabolism of Residual/Peri‐Focally Localized Glioblastoma Cells

**DOI:** 10.1002/ijc.70483

**Published:** 2026-04-09

**Authors:** Frieda Bayler, Jonna Holler, Jacqueline Clüver, Levi Johanning, Dana Hellmold, Nils Oliver Schröder, Jessica Nojszewski, Hajrullah Ahmeti, Carolin Kubelt‐Kwamin, Michael Synowitz, Janka Held‐Feindt

**Affiliations:** ^1^ Department of Neurosurgery University Medical Center Schleswig‐Holstein (UKSH) Kiel Germany

**Keywords:** carbohydrate metabolism, glioblastoma, mechanical high‐intensity focused ultrasound

## Abstract

Glioblastomas (GBMs) are highly aggressive and therapy‐resistant brain tumors, mainly driven by stem‐like cells and profound metabolic plasticity. Novel treatment strategies, including mechanical high‐intensity focused ultrasound (mFUS), are being developed, but their effects on tumor metabolism remain poorly understood. To address this gap, we investigated the impact of mFUS on carbohydrate metabolism in patient‐derived GBM organoids and 3D glioma stem‐like cell (GSC) cultures. We showed that mFUS selectively induced the expression of glycolysis‐ and metabolite‐transport‐associated molecules (GLUT1, HK2, PKM2, LDHA, MCT1, MCT4), particularly in GSCs, as confirmed by qPCR and immunofluorescence. Functional assays demonstrated increased glucose uptake after mFUS, while lactate production remained unchanged. Notably, pharmacological inhibition of GLUT1 or MCT1 potentiated the cytotoxic effects of mFUS, significantly reducing the survival of peri‐focal GSCs. Together, these results reveal that mFUS promotes metabolic adaptations in GBM cells and that combined metabolic inhibition may enhance its therapeutic efficacy.

## Abstract (highlights)

What's new?

Glioblastoma (GBM) is highly aggressive and therapy‐resistant, warranting exploration of novel treatment strategies, among them mechanical high‐intensity focused ultrasound (mFUS), which holds significant promise for tumor ablation. The present study examined mFUS‐induced changes specifically in carbohydrate metabolism, which affects vulnerability to mFUS, in patient‐derived GBM organoids and glioma stem‐like cell (GSC) cultures. Analyses show that mFUS increased expression of glycolysis and metabolite transport markers and enhanced glucose uptake, particularly in GSCs, without altering lactate production. Inhibiting GLUT1 or MCT1 significantly enhanced mFUS‐induced cytotoxicity. The findings suggest that combining mFUS with metabolic inhibitors may improve therapeutic effectiveness against GBM.

## Graphical abstract

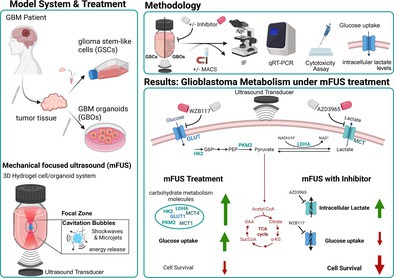

**Abbreviations**
AIP: average incident power
GBM: glioblastoma
GBO: glioblastoma organoids
GLUT1: glucose transporter 1
GSC: glioma stem‐like cell
HK2: hexokinase 2
LDHA: lactate dehydrogenase A
MACS: magnetically activated cell sorting
MCT1/4: monocarboxylate transporters 1/4
mFUS: mechanical high‐intensity focused ultrasound
MSI1: Musashi (Drosophila) homolog 1
OCT4: octamer‐binding transcription factor 4
PKM2: pyruvate kinase M2
SOX2: sex‐determining region Y‐box 2

## 1 Introduction

Glioblastomas (GBMs) are aggressive primary brain tumors that grow rapidly and infiltrate surrounding tissue. Despite advances in surgery and treatment, prognosis remains poor due to early relapse and resistance [1], mainly caused by their high genetic and cellular heterogeneity, which leads to distinct subtypes and adaptable tumor cell subpopulations [2, 3, 4, 5, 6]. Besides the cellular heterogeneity of GBM, various metabolic changes are observed that play a vital role in GBM malignancy and progression [7]. Indeed, tumor cells adapt to their environment by reprogramming their cellular metabolism, allowing them to grow, survive, and proliferate in a constantly changing environment [8]. In this context, aerobic glycolysis, known as the “Warburg effect,” appears to be particularly important [7]. This metabolic shift is characterized by increased glucose uptake and consumption, leading to higher lactate production even when oxygen is present. The Warburg effect occurs due to the increased transcription of genes that encode glucose transporters and enzymes involved in glycolysis, making them potential targets for therapy [8].

To address GBMs' adaptive and heterogeneous behavior, many therapeutic strategies have been explored [9, 10], but none have led to significant improvements or cures. Given the limited efficacy of current therapies, new approaches are being developed to control tumor growth, eradicate cancer cells, and treat unresectable tumors [11]. In this context, transcranial focused ultrasound (FUS) has recently attracted attention as a non‐invasive modality for tumor ablation and therapy. FUS is an innovative technology that facilitates the precise and controlled application of ultrasound energy to intracranial targets, both spatially and temporally. Its applications in GBMs are varied and include, for instance, high‐intensity thermal ablation of tumor tissue, low‐intensity transient disruption of the blood–brain barrier to improve drug delivery, or activation of chemical agents known as sonosensitizers that induce cell death [12, 13, 14, 15, 16, 17, 18, 19, 20].

Mechanical high‐intensity focused ultrasound (mFUS) has emerged as a promising modality for tissue ablation. Unlike thermal FUS, which utilizes prolonged sonication to generate heat‐induced lesions of millimeter dimensions, often resulting in unintended damage to healthy tissue [21, 22], mFUS employs brief ultrasound pulses to minimize heating during the ablation process [13, 15]. The primary mechanism of mFUS involves acoustic cavitation, wherein pressure gradients induced by ultrasound cause microbubbles within the tissue to grow, oscillate, and collapse [13, 15]. This phenomenon, known as inertial cavitation, occurs under high sound pressure conditions and leads to disruption of cell membranes and tissue destruction while preserving surrounding healthy tissue [21, 22, 23, 24]. Recent studies indicate that mFUS ablation presents a promising therapeutic option for malignant brain tumors and is regarded as a vital future treatment modality for such tumors [22, 23, 24, 25].

Nevertheless, given that the currently available in vivo mFUS systems for rodents, designed for the mechanical ablation of brain tumors, are largely based on specialized custom‐made preclinical setups, and considering that rodent models have constrained translational potential due to different microenvironmental conditions and, thus, discrepancies in GBM biology, the influence of mFUS on, for example, residual or peri‐focally localized GBM cells remains largely unexamined [26, 27]. Consequently, we have developed an in vivo‐adapted in vitro mFUS system to investigate the effects of mFUS on residual/peri‐focal GBM cells within specific patient‐derived GBM organoids (GBOs) and 3D‐cultured GBM cells [28]. Utilizing this system, we recently demonstrated that mFUS impacts the stemness and dormancy characteristics of residual GBM cells, ultimately leading to enhanced survival of these cells following subsequent chemotherapy [28]. Furthermore, our investigation revealed that mFUS resulted in a higher number of dying glioma stem‐like cells (GSCs) with increasing average incident power (AIP). Moreover, in GBOs, a higher expression of activated/cleaved (c)Caspase 3 became detectable, especially near the focal region of GBOs. Reactive oxygen species appeared after mFUS in vitro, and mechanoreceptor expression increased in GSCs. Additionally, mFUS activated the PI3‐kinase/Akt signaling pathway in GBM cells [28]. However, since therapy resistance and recurrence in GBMs are also influenced by metabolic changes, understanding how mFUS impacts these factors is essential. Therefore, this study aims to elucidate the effects of especially mFUS as a promising acoustic cavitation‐based technology on carbohydrate metabolism in residual/peri‐focal GBM cells after treatment in patient‐derived GBOs and 3D‐cultured GBM cells.

## 2 Materials and Methods

### 2.1 Preparation of Patient‐Derived GBM Organoids

Human GBM tissue samples were obtained from surgical resections at the Department of Neurosurgery, University Medical Center Schleswig‐Holstein, Kiel, Germany. The tissue was diagnosed and classified as IDH wild‐type GBMs, CNS WHO grade 4, by a neuropathologist according to WHO criteria at the University Medical Center Hamburg‐Eppendorf, Germany. GBOs were prepared following Jacob et al.'s protocol and cultured with GBO medium (consisting of 50% DMEM/F12 (Thermo Fisher Scientific, Waltham, MA, USA), 50% Neurobasal medium (Thermo Fisher Scientific), GlutaMAX, nonessential amino acids (Thermo Fisher Scientific), penicillin–streptomycin, N2 and B27 supplements without vitamin A (Thermo Fisher Scientific), 2‐mercaptoethanol (Thermo Fisher Scientific), and human insulin (Sigma‐Aldrich, St. Louis, MO, USA)) without growth factors or fetal calf/horse serum as described [28]. The GBOs were previously characterized by our group [28].

### 2.2 Cultivation of Patient‐Derived Glioma Stem‐Like Cells

GSCs were obtained from patient samples by dissociating tumor tissue collected during surgeries at the Department of Neurosurgery, University Medical Center Schleswig‐Holstein, Kiel, Germany. GSCs were cultured under stem‐like conditions in F12 media supplemented with B27 supplement (Thermo Fisher Scientific) and 1% penicillin–streptomycin (10,000 U/mL), 10 ng/mL epidermal growth factor (Peprotech, Cranbury, NJ, USA), and 10 ng/mL basic fibroblast growth factor (Immunotools, Friesoythe, Germany). GSCs were confirmed by their ability to form neurospheres, survive, proliferate in stem cell media, and differentiate into more mature cell types, as previously validated [29, 30, 31, 32]. The purity was verified through immunostaining with cell type‐specific markers and by confirming the absence of mycoplasma contamination. All experiments were performed with mycoplasma‐free cells.

### 2.3 3D Hydrogel GBO/GSC Cultures for In Vitro mFUS Application

For 3D hydrogel cultures, VitroGel IKVAV‐Hydrogel (The Well Bioscience, Monmouth Junction, NJ, USA) was used at a 1:5 dilution, matching the brain tissue stiffness [33], and prepared according to the manufacturer's instructions. To perform in vitro mFUS treatment, GBO/GSC samples were assembled directly in Covaris tubes (1 mL; Covaris LLC., Woburn, Massachusetts, USA). For GBO 3D hydrogel cultures, about 30 GBOs were transferred into the tubes, and their medium was aspirated. The GBO medium was mixed with hydrogel at a 4:1 ratio and added to GBOs in the tubes. After polymerization, the hydrogel was covered with GBO medium and cultured for 24 h before mFUS as previously described [28]. For GSC cultures, the same procedure was used with the prepared Covaris tubes and IKVAV hydrogel, containing 24 × 10^6^ GSCs/mL. The hydrogel was covered with GSC growth medium and cultured for 24 h before mFUS, as previously described [28].

### 2.4 In Vitro Mechanical Focused Ultrasound (mFUS) Setup

The M220 ultrasound generator, equipped with a 0.5‐MHz solid‐state ultrasound transducer and geometrically focused acoustic energy, was used (Covaris LLC.; https://www.covaris.com/technology/afa‐technology) [28]. The device was designed as a rapid and efficient platform for FUS‐induced mechanical preparation of biological samples. Covaris states that the system produces a wavelength of a few millimeters, focusing ultrasound energy in a sample vessel within a water bath. Focused ultrasonic bursts control cavitation bubbles in an isothermal, non‐contact environment, preventing thermal damage. These bubbles oscillate or collapse to generate shear stresses in the sample. The system allows customization of peak incident power (PIP), duty cycle (DF), cycles per burst (CPB), and duration to match required settings while maintaining temperature. It also accommodates spherical reaction vessels up to 1 mL. The mFUS settings were as follows based on previous investigations [28]: AIP 11 W: PIP 55 W, DF 20%, CPB 200, 10 s for patient‐derived GBOs; AIP 15 W: PIP 75 W, DF 20%, CPB 200, 10 s for patient‐derived GSCs. The validation of the in vitro mFUS setup was previously described by our group [28].

### 2.5 Quantitative Polymerase Chain Reaction (qPCR)

To evaluate regional gene expression differences in GBOs or GBM cell cultures after mFUS treatment (AIP 11 or 15 W), the respective 3D hydrogel GBO/cell cultures were snap‐frozen in liquid nitrogen and removed from Covaris tubes for sectioning, ensuring sample orientation remained intact for regional analysis. Serial sections were prepared at −20°C using a cryostat (#CM 1100, Leica Biosystems, Nussloch, Germany) for RNA isolation and immunofluorescence of sections from focal and peri‐focal regions [28]. RNA was isolated with TRIzol (Invitrogen, Carlsbach, CA, USA) or ARCTURUS PicoPure kits (Applied Biosystems, Forster City, CA, USA), following manufacturer instructions. DNase digestion, cDNA synthesis, reverse transcription, and qPCR were performed as previously described [29, 30, 31, 32] using TaqMan primer probes. Gene‐specific primers and probes are listed in Table S1. The threshold cycles (C_T_) were determined, and the ∆C_T_ values of each sample were calculated as the C_T_ of the gene of interest minus the C_T_ of glyceraldehyde‐3‐phosphate dehydrogenase (GAPDH). Samples with undetectable expression were excluded from mean expression calculations. Gene expression regulation upon mFUS treatment is shown as relative gene expression, calculated as n‐fold expression changes = $2^{∆C_{T}(control)-∆C_{T}(stimulus)}$.

### 2.6 Immunofluorescence

For staining, cryostat sections were incubated overnight at 4°C with primary antibodies, followed by a 1 h incubation with secondary antibodies at 37°C and nuclear staining as described previously [29, 31, 32]. The embedded slides were analyzed using fluorescence microscopy (AxioObserver.Z1; Carl Zeiss AG, Oberkochen, Germany) with ZEN 3.5 (blue edition) software (Carl Zeiss AG). The primary antibodies used are listed in Table S2. When primary antibodies were from the same species, nonspecific binding was blocked with species‐specific F(ab) fragments (1:1000, from Jackson ImmunoResearch, West Grove, PA, USA). For negative controls, primary antibodies were omitted. Secondary antibodies included donkey anti‐mouse or anti‐rabbit IgG labeled with Alexa Fluor 488 or Alexa Fluor 555 (1:1000; Thermo Fisher Scientific). Our Figures include uncropped immunofluorescence images with accompanying detailed images shown in the corresponding sizes.

### 2.7 Cytotoxicity Assay

After mFUS treatment, cells were recovered from the 3D hydrogels using Cell Recovery Solution (The Well Bioscience) according to the manufacturer's protocol, then subjected to further analysis. The cytotoxic effects were assessed with the CytoTox‐Fluor^TM^ Cytotoxicity Assay (Promega, Madison, WI, USA) following the manufacturer's instructions as described [28, 30]. Supernatants from treated and control cells were collected, and fluorescence was measured using a microplate reader (Infinite M200Pro, TECAN, Zürich, Switzerland) at an excitation wavelength of 485 nm and an emission wavelength of 535 nm. Dead cell numbers were determined using a standard curve generated from digitonin‐lysed cell dilutions (82.5 μg/mL; Merck Millipore, Darmstadt, Germany). The percentage of dead cells was calculated as the ratio of dead to total cells, as previously described [28, 30]. Cell viability and cell numbers were determined by counting viable cells using a hemocytometer at defined time points.

### 2.8 CD11b and CD3 Cell Depletion Using Magnetically Activated Cell Sorting (MACS)

GBOs were collected from the 3D hydrogels 24 h after mFUS treatment (AIP 11 W), and processed using MACS technology to deplete cells expressing CD11b and CD3, including macrophages, microglia, T lymphocytes, and NK cells, as described [28, 30]. Specifically, single‐cell suspensions were prepared from 400 mg of GBOs with the Neural Dissociation Kit (T) (Miltenyi Biotech GmbH, Gladbach, Germany), labeled with CD11b/CD3 MicroBeads (Miltenyi Biotech GmbH), and separated using MACS LS columns according to the manufacturer's protocol. Further analyses were conducted using qPCR.

### 2.9 Determination of Intracellular Lactate Levels

A lactate assay was performed according to the manufacturer's instructions using the L‐Lactate assay kit (ab65331, Abcam, Rozenburg, The Netherlands). Specifically, after homogenizing 0.3 × 10^6^ mFUS (un)treated GSCs (AIP 15 W) with the lactate assay buffer (ab65331, Abcam), endogenous lactate dehydrogenase was removed using the deproteinizing sample preparation kit‐TCA (ab204708, Abcam). Intracellular lactate concentrations (nmol/1.0 × 10^6^ cells) were measured against internal lactate standard curves using a microplate reader to detect optical density at 450 nm (Infinite M200Pro, TECAN).

### 2.10 Determination of Glucose Uptake

A glucose uptake assay was performed according to the manufacturer's instructions using the Glucose Uptake Glo assay kit (J1341, Promega). Specifically, 0.3 × 10^6^ mFUS (un)treated GSCs (AIP 15 W) were incubated with 2‐deoxy‐D‐glucose (1 mM) for 10 min. The cells were lysed, and neutralization buffer was added to the lysate. The samples were then centrifuged, and the luminescence of the homogenates was measured against internal 2‐desoxy‐D‐glucose standard curves using a luminometer with a one‐second integration time (Infinite M200Pro, TECAN).

### 2.11 Inhibition Experiments

The inhibitors AZD3965 (MCT1 Inhibitor, Biomol, Hamburg, Germany; 25–500 nM) and WZB117 (GLUT1 Inhibitor, Merck Millipore; 10–100 μM) were dissolved in ethanol and added to the cell medium at the specified concentrations for individual experiments. In 2D culture, cells were treated with inhibitors for three days. In 3D culture, cells were pre‐stimulated for 24 h, embedded in hydrogels containing an inhibitor, and after a further 24 h, the medium was replaced prior to mFUS application (AIP 15 W). Cells were cultured further in hydrogels for 24 h with an inhibitor. Cell numbers, cytotoxicity, glucose uptake, and intracellular lactate levels were measured on the last day of the experimental design.

### 2.12 Statistical Analysis

The data were analyzed statistically using GraphPad Prism 8.4 software (GraphPad Software, San Diego, CA, USA). All experiments were performed with a minimum of *n* = 3 independent biological replicates, whenever applicable. Samples were only included in the study if they met established internal quality control criteria, such as the absence of mycoplasma contamination and a correct histological diagnosis. The sample size is specified in the figure legends. Depending on the experimental design, a two‐tailed Student's *t*‐test or a one‐way ANOVA with Dunnett's multiple comparisons post hoc test was performed, as indicated in each figure caption. The data fulfilled the preconditions of the tests, and the variance between the statistically compared groups was similar. Statistical significance is indicated by asterisks based on the *p*‐value: *p < 0.05, **p < 0.01, and ***p < 0.001.

## 3 Results

### 3.1 mFUS Regulates the Expression of Metabolism‐Related Molecules in GBOs in a Region‐ and Marker‐Dependent Manner

First, we examined the effect of mFUS (AIP 11 W; 24 h post‐mFUS) on patient‐derived GBOs grown in 3D hydrogels. The characterization and quality control of the GBOs, as well as the in vitro mFUS setup used, have been described previously [28]. The molecules analyzed included various metabolism‐related transporters and enzymes, specifically glucose transporter 1 (GLUT1), hexokinase 2 (HK2), pyruvate kinase M2 (PKM2), lactate dehydrogenase A (LDHA), and monocarboxylate transporters 1 and 4 (MCT1 and MCT4) [7, 8, 32]. Notably, mFUS‐treated samples labeled as margins were GBOs from peri‐focal regions of the entire GBO‐containing samples, while samples labeled as the center were from the focal area of mFUS (surviving cells). Samples labeled as controls did not receive mFUS treatment.

**FIGURE 1:**
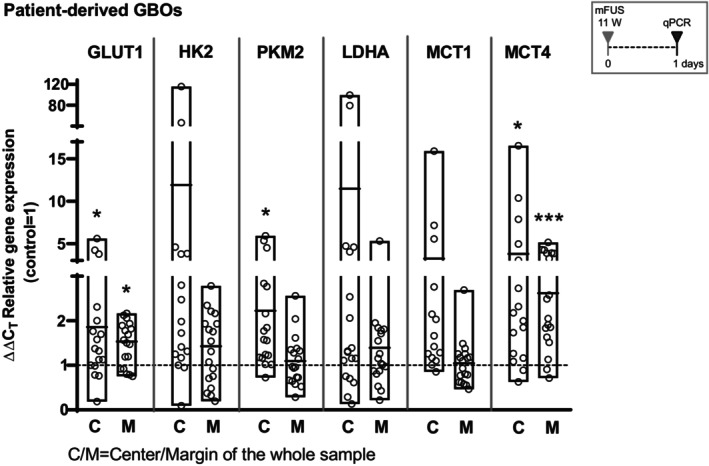
GBOs treated with mFUS (average incident power 11 W) showed an increased mRNA expression of carbohydrate metabolism‐associated markers in the residual/peri‐focal cells in a complex marker‐ and region‐dependent manner (center/margin of whole samples) compared to average unstimulated controls (control = 1). *n* = 10 biological replicates with *n* = 1–2 technical replicates each. GLUT1, glucose transporter 1; HK2, hexokinase 2; LDHA, lactate dehydrogenase A; MCT1, monocarboxylate transporter 1; MCT4, monocarboxylate transporter 4; PKM2, pyruvate kinase M2. Statistical differences compared to untreated controls were determined using a two‐tailed Student's *t*‐test and are indicated above the bars (*p < 0.05; ***p < 0.001). Horizontal lines represent the mean of the data set.

An initial quantitative PCR (qPCR) analysis revealed that the various GBOs exhibited a mFUS‐promoted regulation of mRNA expression related to metabolic molecules. The majority of cases demonstrated an induction of these molecules, particularly in the focal region (center). Due to the heterogeneity among the GBOs, the effects varied; however, they were generally observable (Figure 1).

**FIGURE 2:**
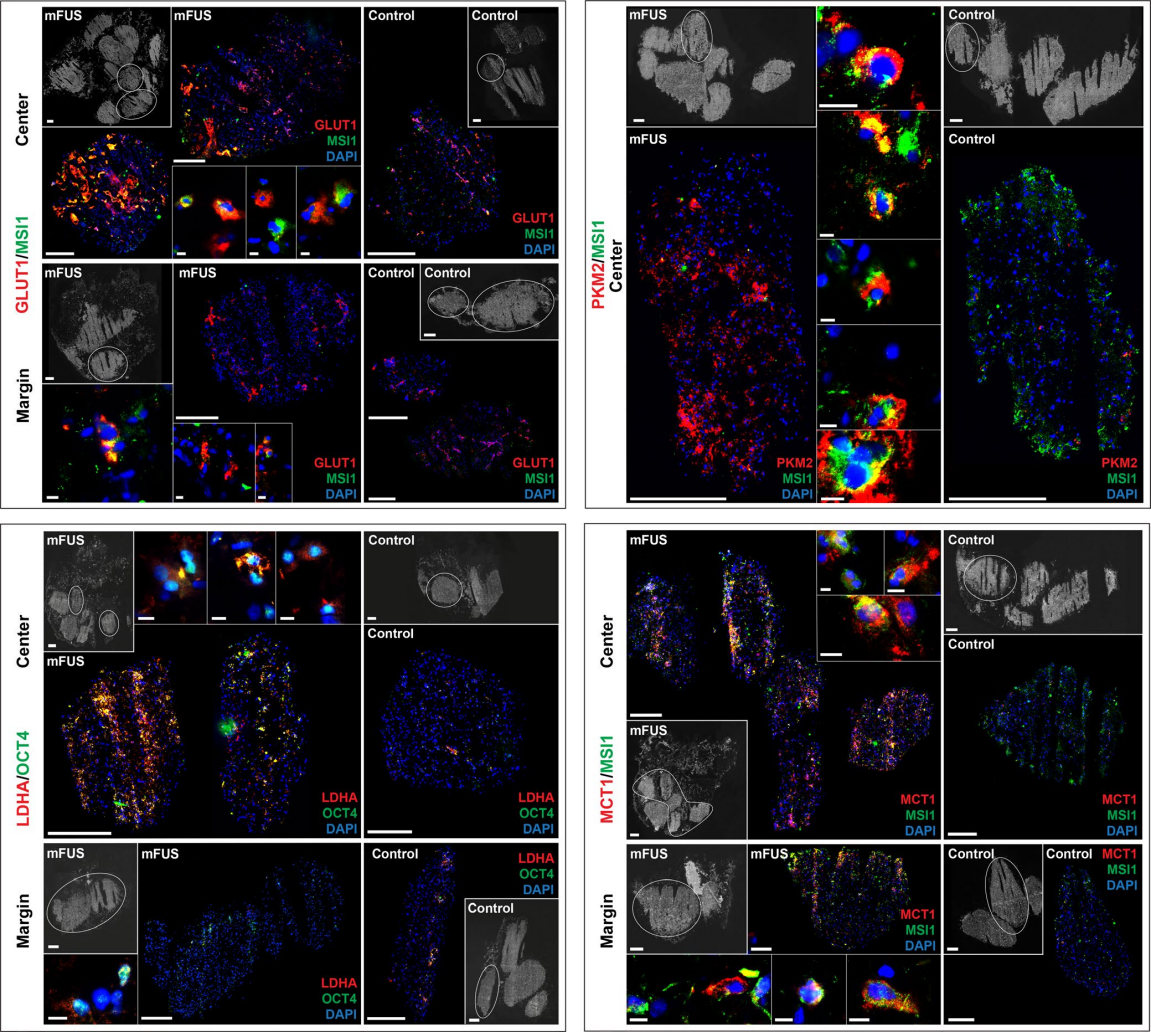
GBO‐containing samples were treated with mFUS (average incident power 11 W). They were further cultured for 24 h, cryofixed, and serially sectioned. Metabolism‐associated markers (GLUT1, PKM2, LDHA, MCT1) were co‐stained with stem‐like cell markers (MSI1, OCT4) in sections from the sample's focal (center) and peri‐focal (margin) mFUS regions in comparison to untreated controls. Bar: 400 μm (GBO samples and whole sections), 10 μm (inserts). Circled GBOs in whole sections show those GBOs that were visualized in detail in the immunofluorescence. *n* = 3 different GBO preparations. DAPI, 4′,6‐diamidino‐2‐phenylindole; MSI1, Musashi (Drosophila) homolog 1; OCT4, octamer binding transcription factor 4. For further abbreviations, please refer to the previous Figures.

**FIGURE 3:**
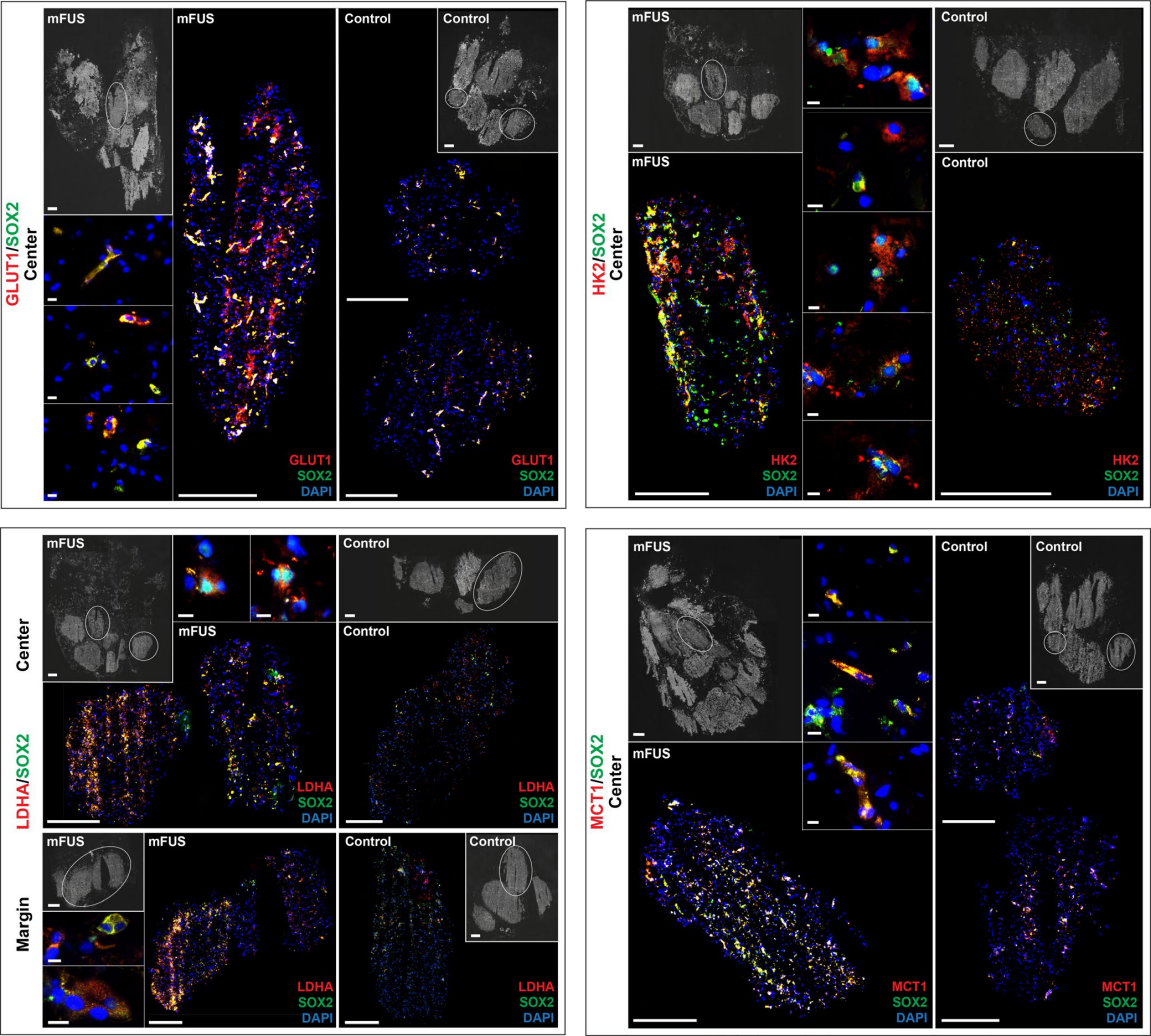
Multi‐Immunofluorescence staining was performed on mFUS‐treated (average incident power 11 W) GBO‐containing samples cultured for 24 h after mFUS in comparison to untreated controls. The samples were cryofixed and serially sectioned. Metabolism‐associated markers (GLUT1, HK2, LDHA, MCT1) were co‐stained with the stem‐like cell marker SOX2 in sections from the sample's focal (center) and peri‐focal (margin) mFUS regions. Bar: 400 μm (GBO samples and whole sections), 10 μm (inserts). Circled GBOs in whole sections show those GBOs that were visualized in detail in the immunofluorescence. *n* = 3 different GBO preparations. SOX2, sex‐determining region Y‐box 2. For further abbreviations, please refer to the previous Figures.

**FIGURE 4:**
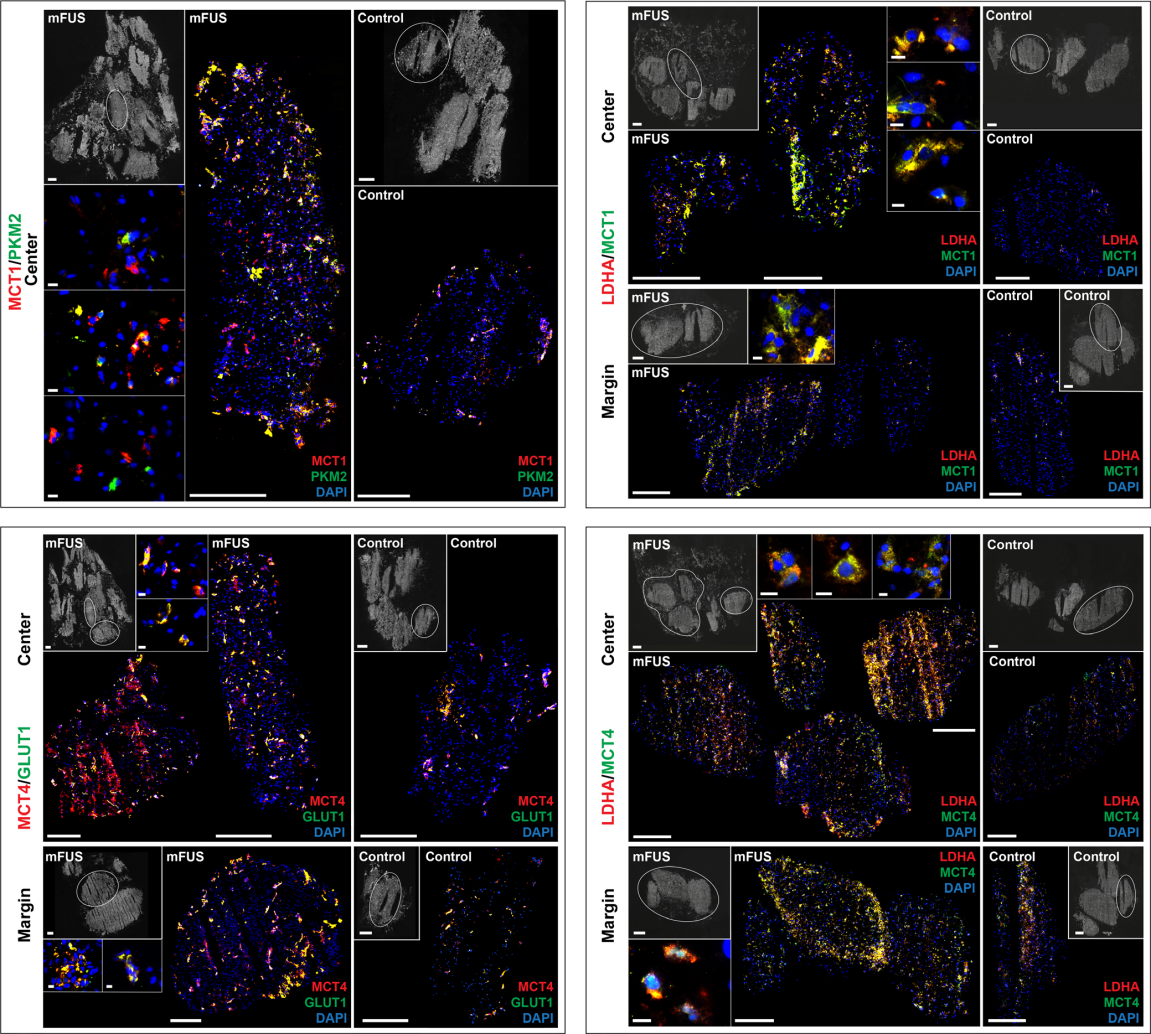
mFUS (average incident power 11 W) treated GBO‐containing samples were further cultured for 24 h, cryofixed and serially sectioned. In sections from the sample's focal (center) and peri‐focal (margin) mFUS regions, metabolism‐associated markers (GLUT1, PKM2, LDHA, MCT1, MCT4) were co‐stained using multi‐immunofluorescence in comparison to untreated controls. Bar: 400 μm (GBO samples and whole sections), 10 μm (inserts). Circled GBOs in whole sections show those GBOs that were visualized in detail in the immunofluorescence. *n* = 3 different GBO preparations. For abbreviations, please refer to the previous Figures.

To get an initial indication of the cellular source of mFUS‐induced metabolic molecule expression, immunofluorescence multi‐staining was performed on sections taken from the center or the margin of the entire, mFUS‐(un)treated GBO‐containing samples (AIP 11 W; 24 h post‐mFUS). Since GSCs are the main contributors to GBM development and recurrence [4], special attention was given to co‐staining metabolism‐related molecules with stemness‐associated markers such as sex‐determining region Y‐box 2 (SOX2), Musashi (Drosophila) homolog 1 (MSI1), and octamer‐binding transcription factor 4 (OCT4) [28, 29, 30, 31, 32, 34], as well as to the co‐staining of metabolic molecules with each other (Figures 2, 3, 4).

The immunohistochemical examinations confirmed the qPCR results. An increase in metabolic markers was observed after mFUS, with the effect mostly more pronounced in the focal area. Co‐staining of metabolic markers with stemness‐related molecules was observed in all combinations, with individual positive cells for each marker (Figures 2 and 3). Notably, LDHA co‐stained with MCT1 and MCT4 in the center and margin of treated GBO samples, more so than in controls. Similarly, GLUT1 co‐stained with MCT4, and PKM2 with MCT1, showing stronger expression in treated samples, although less pronounced than the LDHA combinations (Figure 4).

### 3.2 mFUS Promotes the Expression of Metabolism‐Related Molecules in Patient‐Derived Glioma Stem‐Like Cells

**FIGURE 5:**
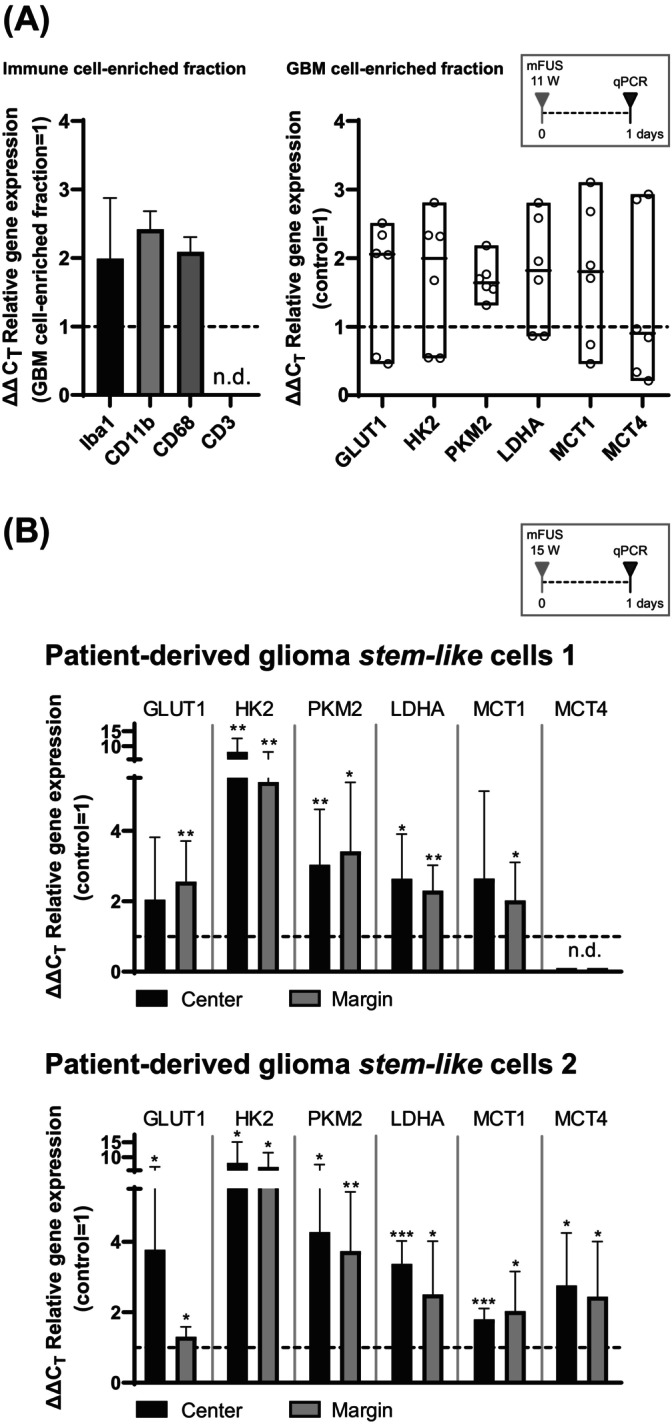
(A) GBO‐containing samples were treated with mFUS (average incident power 11 W) and further cultured for 24 h. Depletion of immune cells was performed using CD11b/CD3 magnetically activated cell sorting (MACS). *n* = 1 biological replicate with *n* = 2 technical replicates. *Left*: Exemplary data on the success of MACS‐based immune cell depletion is shown. *Right*: mRNA expression of metabolism‐associated markers of the GBM‐cell enriched fraction showed an increase in GBO‐containing samples treated with mFUS compared to average unstimulated controls (control = 1). *n* = 3 biological replicates with *n* = 1–2 technical replicates each. Horizontal lines represent the median of the data set. (B) Two different preparations of patient‐derived glioma stem‐like cells (GSCs) were treated with mFUS (average incident power 15 W) and further cultured for 24 h after mFUS treatment. The induction of the mRNA expression of metabolism‐associated markers could be determined in residual/peri‐focal cells compared to average unstimulated controls (control = 1). Focal (center) and peri‐focal (margin) mFUS regions of whole samples were analyzed separately. GSC1: *n* = 3–5 biological replicates with two technical replicates each; GSC2: *n* = 3 biological replicates with 1–2 technical replicates each. Significant differences compared to the untreated controls were determined by a two‐tailed Student's *t*‐test and are indicated directly above the bars (*p < 0.05; **p < 0.01; ***p < 0.001). Error bars correspond to the standard deviation. CD, cluster of differentiation, IBA1, calcium‐binding adapter molecule 1; n.d., non‐determined. For further abbreviations, please refer to the previous Figures.

To support the findings concerning the cellular source of increased expression of metabolism‐related markers after mFUS, exemplary 3D hydrogel GBO preparations with and without mFUS (AIP 11 W; 24 h post‐mFUS) were processed, followed by depletion of CD11b‐ and CD3‐positive immune cells using magnetically activated cell sorting (MACS). After confirming immune cell removal (Figure 5A, left) to ensure that we can attribute the observed regulation to the tumor cell subpopulation, analysis of mRNA expression of metabolic markers revealed an increase in nearly all of these to varying degrees (~2‐fold on average, except for MCT4) in the GBM cell‐enriched fraction after mFUS (Figure 5A, right).

To further validate the findings of the GBOs, 3D hydrogel cultures of patient‐derived GSCs were exposed to mFUS (AIP 15 W; 24 h post‐mFUS). Two independent GSC preparations were used (Figure 5B). In fact, mFUS caused the expression of all the metabolic markers examined in the GSCs to increase. The most significant increase was observed for HK2 (~8‐ to 10‐fold). The other markers showed a ~2‐ to 4‐fold rise after mFUS, although MCT4 was not detected in the patient‐derived GSC1 culture. Interestingly, the induction of various markers in the margin and center after mFUS was similar, except for GLUT1 in the GSC2 culture, which showed a significantly greater increase in the center.

### 3.3 Inhibition of Specific Metabolic Markers Enhances the mFUS Effect in Patient‐Derived Glioma Stem‐Like Cells

Given that a change in metabolism in tumor cells serves as a survival strategy [7, 8, 32], we then examined whether the inhibition of specific metabolic molecules, specifically GLUT1 using WZB117 and MCT1 using AZD3965, could enhance the effect of mFUS. After determining the optimal concentrations of the respective inhibitors (effective inhibition of glucose uptake and lactate export without significantly affecting growth rate or cell death: WZB117: 10 μM; AZD3965: 25 nM; Figure S1), we treated two different patient‐derived 3D hydrogel GSC cultures with the respective inhibitors beginning 48 h before mFUS and maintained throughout the procedure until 24 h after treatment (AIP 15 W). We then analyzed cell numbers, cell death, glucose uptake, and intracellular lactate levels in the GSCs compared to the respective unstimulated controls.

**FIGURE 6:**
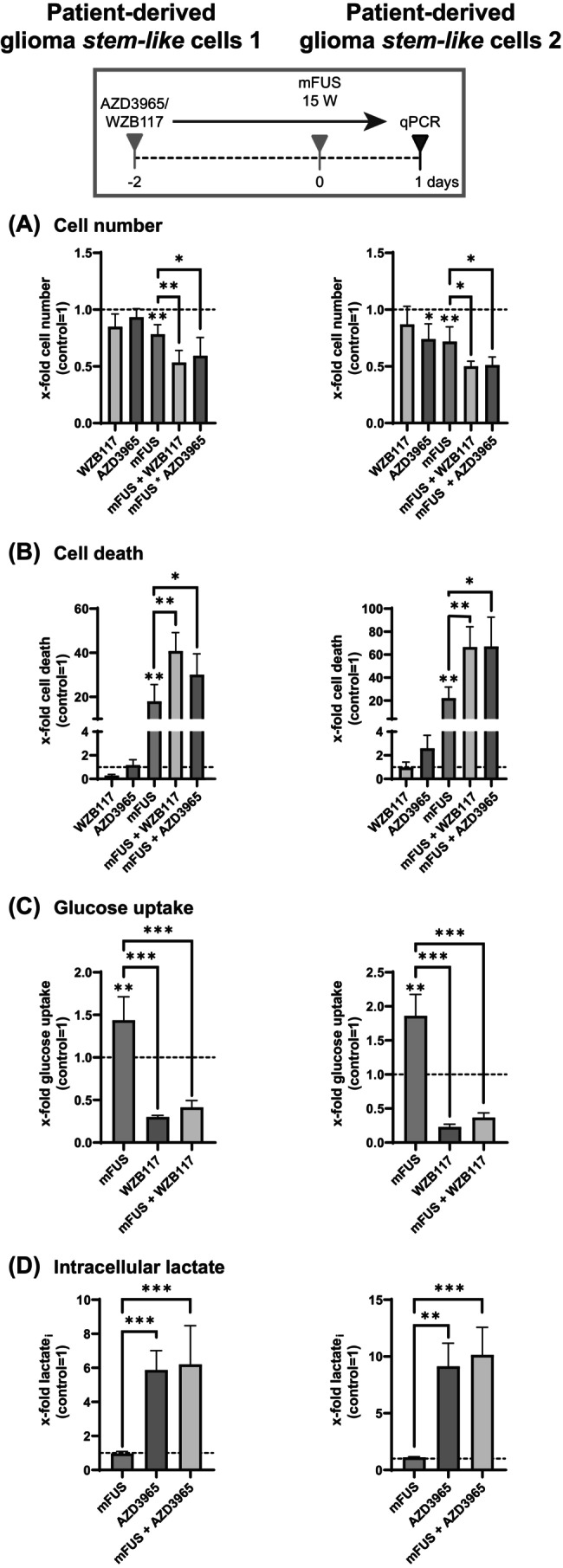
Two different preparations of patient‐derived glioma stem‐like cells (GSCs) were stimulated with GLUT1 inhibitor WZB117 (10 μM) or MCT1 inhibitor AZB3965 (25 nM) for 48 h before treatment with mFUS (average incident power 15 W). They were further cultured for 24 h with continued inhibitor stimulation. x‐fold inductions are each shown in comparison to average controls (control = 1). (A) Recovered cell numbers 24 h after mFUS. GSC1: *n* = 3–8 biological replicates with one technical replicate each; GSC2: *n* = 3–6 biological replicates with one technical replicate each. (B) Cell death rates 24 h after mFUS treatment. GSC1: *n* = 2–8 biological replicates with two technical replicates each; GSC2: *n* = 2–5 biological replicates with two technical replicates each. (C) Effect of mFUS treatment and inhibitor stimulation on glucose uptake of GSCs. GSC1, GSC2: *n* = 3–4 biological replicates with two technical replicates each. (D) Intracellular lactate amounts 24 h after mFUS treatment. GSC1: *n* = 5 biological replicates with two technical replicates each; GSC2: *n* = 3–4 biological replicates with two technical replicates each. Significant differences compared to the untreated controls were determined by a two‐tailed Student's *t*‐test and are indicated directly above the bars. Statistical significance of differences comparing mFUS treatment alone with combined treatment was determined using a one‐way ANOVA with Dunnett's multiple comparisons post hoc test and is indicated above horizontal lines (*p < 0.05; **p < 0.01; ***p < 0.001). Error bars correspond to the standard deviation. For abbreviations, please refer to the previous Figures.

Both WZB117 and AZD3965 further enhanced a statistically significant mFUS‐regulated reduction in cell numbers in both 3D hydrogel GSC cultures (Figure 6A). Specifically, the number of cells from the hydrogels after mFUS treatment with concurrent inhibitor was approximately half that of the untreated controls. In contrast, mFUS treatment alone resulted in only a ~0.25‐fold reduction in cell numbers in both GSCs studied. The observed cell death rates also supported these findings (Figure 6B). mFUS alone caused approximately a 20‐fold increase in the death rate compared to untreated controls. When combined with WZB117, the effect was more than twice as high, leading to a statistically significant 41‐fold increase in GSC1 and a 67‐fold increase in GSC2. AZD3965, combined with mFUS, increased the cell death rate 30‐fold in GSC1 and 67‐fold in GSC2 compared to the untreated control (all statistically significant compared to mFUS treatment alone).

An analysis of glucose uptake and intracellular lactate levels (Figure 6C,D) showed that mFUS alone could increase glucose uptake by approximately 1.5–2‐fold in the 3D GSC cultures. In contrast, mFUS alone did not influence intracellular lactate levels. The combined treatment with mFUS and WZB117 reduced glucose uptake to about 0.25‐fold that of untreated controls. AZD3965, when combined with mFUS, caused a sixfold increase in intracellular lactate levels in GSC1 and roughly a tenfold increase in GSC2 (all statistically significant compared to mFUS alone). The use of the respective inhibitors alone produced similar results.

## 4 Discussion

FUS represents a contemporary, non‐invasive modality employed for both diagnostic and therapeutic objectives [21, 22]. Aside from the more widely recognized FUS techniques, another tissue ablation mechanism, namely mechanical high‐intensity FUS (mFUS), is gaining increasing prominence [12, 13, 14, 15, 16, 17, 18, 19, 20, 21, 22]. Indeed, mFUS‐induced ablation has demonstrated favorable outcomes in the treatment of various solid tumors, with Phase I clinical trials confirming its safety and efficacy in managing liver tumors, benign prostatic hyperplasia, and aortic valve calcification [23, 24, 25]. Additionally, recent investigations have indicated that mechanical ablation constitutes a promising modality for the treatment of malignant brain tumors [23, 24, 25].

Since altered tumor metabolism involves the Warburg effect, promoting GBM malignancy [7, 32], and research on how mFUS impacts the metabolic profile of surviving or peri‐focally located tumor cells is lacking, this study examined the influence of mFUS on GBM carbohydrate metabolism. We demonstrated that mFUS induced the expression of the key molecules GLUT1, HK2, PKM2, LHDA, MCT1, and MCT4 involved in carbohydrate metabolism in GBOs, mainly in the surviving cells within the focal region, and to a lesser extent in the peri‐focal regions. We also traced the cellular source of this upregulation to GSCs. Because this upregulation might be a tumor cell's countermeasure to survive [35, 36], we further tested whether inhibiting GLUT1 and MCT1 could boost the effects of mFUS. Indeed, this inhibition increased the cytotoxic impact of mFUS, accompanied by a reduction in the number of surviving/peri‐focal GSCs. The mFUS‐regulated increase in glucose uptake was also efficiently inhibited by the simultaneous administration of the GLUT1 inhibitor WZB117, whereas mFUS appeared not to affect the lactate export.

Indeed, key factors in altered carbohydrate metabolism, including HK2, PKM2, LDHA, and several transporters such as GLUT1, MCT1, and MCT4 [7, 32, 37, 38], are overexpressed and linked to lower survival in GBMs, participating in tumorigenic processes beyond carbohydrate metabolism [32, 38]. Given the mFUS‐induced upregulation of key molecules involved in carbohydrate metabolism and the enhanced glucose uptake in GSCs, it can be suggested that mFUS supported glycolytic metabolism in our setup. Indeed, although GSCs often prefer oxidative phosphorylation [39], they exhibit metabolic heterogeneity and can switch between oxidative phosphorylation and aerobic glycolysis [32, 40]. These findings are novel, as few studies have investigated the effect of FUS on tumor cell metabolism. Hackett et al. [41] found higher lactate, bicarbonate, and glycerate delivery in FUS‐treated brain areas after temporarily opening the blood–brain barrier with low‐intensity FUS. Conversely, Papadakis et al. [42] observed no changes in glucose metabolism after low‐intensity FUS in rats. Lee et al. [43] examined the peri‐focal zone in a murine model, where areas receiving sublethal hyperthermia initially showed reduced metabolism. After a few days, some cases showed an increase in metabolism beyond the initial level. These results highlighted that FUS has a significant impact on cell metabolism in the peri‐focal zone.

The significance of mFUS in enhancing glycolysis rates as a potential survival strategy is noteworthy, particularly in a clinical context where GSCs may be more susceptible to treatment with glycolytic inhibitors, such as WZB117 or AZD3965. Indeed, combining mFUS with WZB117 or AZD3965 resulted in greater cytotoxic effects and fewer GSCs compared to using mFUS or the inhibitors alone. Additionally, the mFUS‐induced increase in glucose uptake in GSCs was effectively blocked by WZB117. This is especially significant, as some studies have shown that glycolytic activity helps maintain stemness characteristics. Zhao et al. [44] demonstrated that lowering HK2 expression reduced the stemness features of GSCs. Similarly, treatment with the GLUT1 inhibitor WZB117 decreased the expression of stemness markers and lowered the tumor‐forming ability [45]. Others, however, have observed GSCs with high mitochondrial activity, suggesting a more oxidative phenotype [46, 47]. Therefore, combining mFUS with glycolysis inhibitors could be a promising therapeutic strategy for GBMs; however, its success might depend on external factors, such as the tumor microenvironment and cellular stress.

This study demonstrates that, by employing both GBOs and 3D‐cultured primary GSCs, mFUS induces an increase in glycolytic activity in GBM cells. Furthermore, a synergistic effect was observed when mFUS was combined with glycolysis inhibitors WZB117 and AZD3965, resulting in increased cell death. These findings indicate that the augmentation of glycolysis following mFUS treatment represents a survival strategy employed by the remaining or peri‐focal GBM cells.

## 5 Limitation of the Study

Our system is designed exclusively for cavitation‐based mFUS application, which limits direct comparison with FUS modalities driven by other mechanisms (e.g., thermal approaches). At the same time, this specialization strengthens the internal validity and interpretability of the cavitation‐specific effects obtained in this study. In addition, the lack of in vivo validation restricts the broader translational interpretation of our findings. Indeed, rodent in vivo mFUS studies still rely largely on custom‐built setups, as standardized, validated, and commercially available test systems are not yet accessible. Furthermore, the extent to which rodent models capture the heterogeneity of human GBM remains debated. Therefore, we instead prioritized a complex, patient‐derived, human 3D model system. However, it cannot be ruled out that other parameters that may influence the mFUS effect in vivo could not be investigated in the patient‐derived GBOs and 3D‐cultured cells (e.g., migration of other/additional immune cell fractions through an open blood–brain barrier or influence of the surrounding healthy brain tissue). However, given that GBOs are complex, heterogeneous systems and that we made a point of using the intraoperatively obtained materials for mFUS examination as quickly as possible, at least the core findings (regulation of metabolism in the surviving/peri‐focal cells, with GSCs being particularly relevant) should be transferable to the in vivo situation.

## Author Contributions

**Frieda Bayler:** methodology, software, investigation, formal analysis, data curation, visualization, validation, writing – review and editing. **Jonna Holler:** data curation, visualization, methodology, software, validation, formal analysis, investigation, writing – review and editing. **Jacqueline Clüver:** data curation, methodology, software, validation, formal analysis, investigation, writing – review and editing. **Levi Johanning:** formal analysis, investigation, data curation, writing – review and editing. **Dana Hellmold:** methodology, software, investigation, writing – review and editing. **Nils Oliver Schröder:** methodology, investigation, resources, writing – review and editing. **Jessica Nojszewski:** methodology, investigation, writing – review and editing, resources. **Hajrullah Ahmeti:** resources, writing – review and editing. **Carolin Kubelt‐Kwamin:** resources, funding acquisition, writing – review and editing. **Michael Synowitz:** resources, writing – review and editing. **Janka Held‐Feindt:** conceptualization, project administration, writing – original draft, funding acquisition, supervision.

## Funding

The research was funded by the Deutsche Forschungsgemeinschaft/German Research Foundation as part of the Research Training Group “Materials4Brain” (RTG2154; P8) and by a start‐up support grant from the University Medical Center Schleswig‐Holstein, UKSH (2025).

## Ethics Statement

The study was conducted in accordance with the Declaration of Helsinki and was approved by the Ethics Committee of the University of Kiel, Germany (file reference: D524/17).

## Consent

Informed consent was obtained from all subjects involved in the study.

## Conflicts of Interest

The authors declare no conflicts of interest.

## Supporting information


**Table S1:** Identifiers of TaqMan assays.
**Table S2:** Antibodies used for immunohistochemistry.
**Figure S1:** 2D cultures of two preparations of patient‐derived glioma stem‐like cells (GSCs) were stimulated with WZB117 or AZD3965 for 3 days. The growth rate (A), cell death (B), glucose uptake (C) and intracellular lactate (D) were determined under inhibitor stimulation to define the optimal inhibitor concentration. x‐fold inductions are each shown in comparison to average controls (control = 1). *n* = 1–4 biological replicates with 1–2 technical replicates each.

## Data Availability

All data on which the paper's conclusions are based are available to readers in the manuscript. Further information is available from the corresponding author upon request.
