# Supplementary material for: Focused Ultrasound‐Induced Mechanical Ablation Affects the Carbohydrate Metabolism of Residual/Peri‐Focally Localized Glioblastoma Cells
Source: Int J Cancer. 2026 Apr 9;159(4):1005–16. doi: 10.1002/ijc.70483 (PMC13284636; doi:10.1002/ijc.70483)
Supplement: Supplementary file 1 — Table S1: Identifiers of TaqMan assays. Table S2: Antibodies used for immunohistochemistry. Figure S1: 2D cultures of two preparations of patient‐derived glioma stem‐like cells (GSCs) were stimulated with WZB117 or AZD3965 for 3 days. The growth rate (A), cell death (B), glucose uptake (C) and intracellular lactate (D) were determined under inhibitor stimulation to define the optimal inhibitor concentration. x‐fold inductions are each shown in comparison to average controls (control = 1). n = 1–4 biological replicates with 1–2 technical replicates each. [file IJC-159-1005-s001.pdf]

Focused ultrasound-induced mechanical ablation affects the carbohydrate metabolism of residual/peri-focally localized glioblastoma cells

Frieda Bayler, Jonna Holler, Jacqueline Clüver, Levi Johanning, Dana Hellmold, Nils Oliver Schröder, Jessica Nojszewski, Hajrullah Ahmeti, Carolin Kubelt-Kwamin, Michael Synowitz, Janka Held-Feindt

Table of contents

SUPPLEMENTARY TABLES 2

Supplementary Table 1: Identifiers of TaqMan assays. 2

Supplementary Table 2: Antibodies used for immunohistochemistry. 3

SUPPLEMENTARY FIGURE 4

Supplementary tables

Supplementary Table 1: Identifiers of TaqMan assays.

**Human**		
CD3	*cluster of differentiation 3*	Hs01062241_m1
CD11b	*cluster of differentiation 11b (integrin alpha M (ITGAM))*	Hs00167304_m1
CD68	*cluster of differentiation 68*	Hs00154355_m1
GAPDH	*glyceraldehyde-3-phosphate dehydrogenase*	Hs99999905_m1
GLUT1	*glucose transporter 1*	Hs000892618_m1
HK2	*hexokinase 2*	Hs00606086_m1
Iba1	*calcium-binding adapter molecule 1*	Hs00610419_g1
LDHA	*lactate dehydrogenase A*	Hs01378790_g1
MCT1	*monocarboxylate transporter 1*	Hs01560299_m1
MCT4	*monocarboxylate transporter 4*	Hs00358829_m1
PKM2	*pyruvate kinase M2*	Hs00761782_s1

Supplementary Table 2: Antibodies used for immunohistochemistry.

Marker	Specificity	Epitope/dilution	Source
GLUT1	*glucose transporter 1*	Polyclonal rabbit-anti-human IgG; 1:200	Abcam, Rozenburg, The Netherlands; Cat. #ab115730
HK2	*hexokinase 2*	Polyclonal rabbit-anti-human IgG; 1:1,300	Thermo Fisher Scientific, Waltham, MA, USA; Cat. #PA5-29326
LDHA	*lactate dehydrogenase A*	Polyclonal rabbit-anti-human IgG; 1:400	Cell Signaling Technology, Danvers, USA; Cat. #3582
MCT1	*monocarboxylate transporter 1*	Polyclonal rabbit-anti-human IgG; 1:400	Thermo Fisher Scientific; Cat. #PA5-72957
MCT4	*monocarboxylate transporter 4*	Polyclonal rabbit-anti-human IgG; 1:400	Bioss Inc., Woburn, Massachusetts; Cat. #BS-2698R
MSI1	*Musashi (Drosophila) homolog 1*	Monoclonal mouse-anti-rat/human IgG; 1:200	R&D Systems, Minneapolis, Canada; Cat. MAB2628
OCT4	*octamer-binding transcription factor 4*	Polyclonal rabbit-anti-human IgG; 1:200	Cell Signaling Technology; Cat. #2750
PKM2	*pyruvate kinase M2*	Polyclonal rabbit-anti-human IgG; 1:250	Cell Signaling Technology; Cat. #4053
SOX2	*sex-determining region Y-box 2*	Polyclonal rabbit-anti-human IgG; 1:200	Santa Cruz Biotechnology, Dallas, Texas; Cat. #sc-20088

Supplementary figure

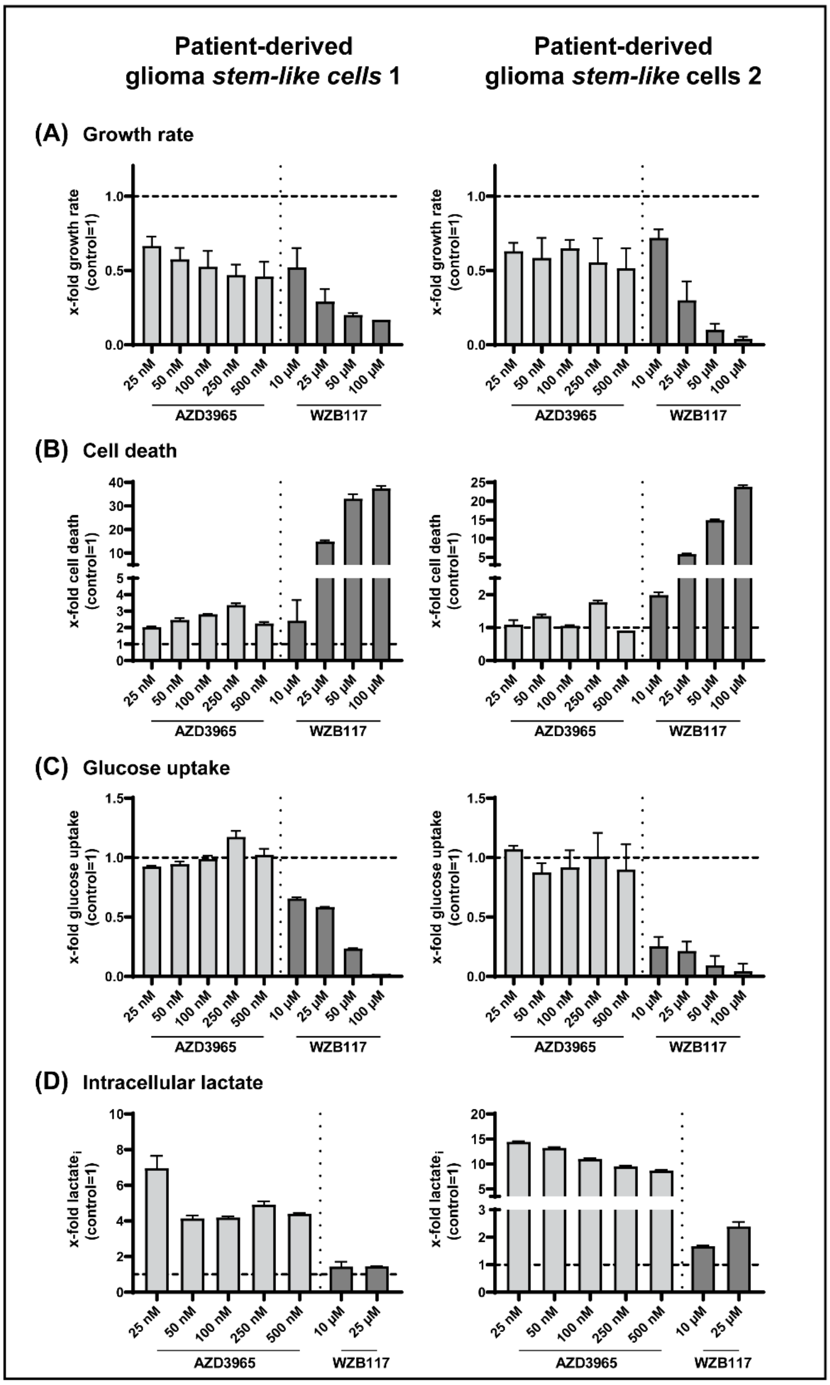

Supplementary Figure 1: 2D cultures of two preparations of patient-derived glioma stem-like cells (GSCs) were stimulated with WZB117 or AZD3965 for 3 days. The growth rate (A), cell death (B), glucose uptake (C) and intracellular lactate (D) were determined under inhibitor stimulation to define the optimal inhibitor concentration. x-fold inductions are each shown in comparison to average controls (control = 1). n=1-4 biological replicates with 1-2 technical replicates each.
